# Dataset on SARS-CoV-2 non-pharmaceutical interventions in Brazilian municipalities

**DOI:** 10.1038/s41597-021-00859-1

**Published:** 2021-03-04

**Authors:** Andreza Aruska de Souza Santos, Darlan da Silva Candido, William Marciel de Souza, Lewis Buss, Sabrina L. Li, Rafael H. M. Pereira, Chieh-Hsi Wu, Ester C. Sabino, Nuno R. Faria

**Affiliations:** 1 Oxford School of Global and Area Studies, University of Oxford, Oxford, UK; 2 Department of Zoology, University of Oxford, Oxford, UK; 3 Virology Research Centre, Ribeirão Preto Medical School, University of São Paulo, Ribeirão Preto, Brazil; 4 Instituto de Medicina Tropical, Faculdade de Medicina da Universidade de São Paulo, São Paulo, Brazil; 5 School of Geography and the Environment, University of Oxford, Oxford, UK; 6 Institute for Applied Economic Research, Brasília, Brazil; 7 Mathematical Sciences, University of Southampton, Southampton, UK; 8 MRC Centre for Global Infectious Disease Analysis, J-IDEA, Imperial College London, London, UK

**Keywords:** Databases, Viral infection

## Abstract

Brazil has one of the fastest-growing COVID-19 epidemics worldwide. Non-pharmaceutical interventions (NPIs) have been adopted at the municipal level with asynchronous actions taken across 5,568 municipalities and the Federal District. This paper systematises the fragmented information on NPIs reporting on a novel dataset with survey responses from 4,027 mayors, covering 72.3% of all municipalities in the country. This dataset responds to the urgency to track and share findings on fragmented policies during the COVID-19 pandemic. Quantifying NPIs can help to assess the role of interventions in reducing transmission. We offer spatial and temporal details for a range of measures aimed at implementing social distancing and the dates when these measures were relaxed by local governments.

## Abstract (summary)

**Table:** 

Measurement(s)	prevention and control intervention • SARS-CoV-2 non-pharmaceutical interventions
Technology Type(s)	Survey
Sample Characteristic - Location	Brazil

Machine-accessible metadata file describing the reported data: 10.6084/m9.figshare.13862108

## Background & Summary

Brazil has seen one of the highest case numbers of COVID-19 in the world. As of 6 December 2020, Brazil recorded over 6,577,177 million cases and more than 176,628 deaths (https://covid19.who.int). SARS-CoV-2 was introduced at least 100 times in Brazil^1^. Non-pharmaceutical interventions (NPIs), although unequal in date of implementation and duration, reduced virus transmission^1,2^. Several factors including changes to the national COVID-19 notification system^3^ and uncoordinated implementation of public health measures may have contributed to rapid epidemic spread across the country. Here, we describe the complexity of asynchronous adoption and easing of NPIs in Brazilian municipalities.

Our data were gathered in a continuous municipal-level survey conducted by the Brazilian Confederation of Municipalities (*Confederação Nacional de Municípios* – CNM). Despite the existing examination of national and state-level NPI strategies^4^, a city-level assessment of NPI’s beyond capitals and second cities^5^ is still missing. Local-level data collection is a challenge because of the number of municipalities in Brazil (5,568 municipalities and the Federal District), and each municipality passed a number of decrees related to COVID-19 control measures. This dataset offers a unique fine-grained understanding of local-level policies in Brazil, aiding future examination on the roles of NPIs on the increase, spread, and duration of local outbreaks.

## Data Sources

The CNM interviewed 4,027 (72.3%) of 5,568 mayors and the Federal District’s government on the implementation and relaxation of NPIs between 13 May and 31 July 2020. Response rates varied by region: North (29.1% of 450 municipalities), Northeast (50.5% of 1,793 municipalities), Centre-West (71.7% of 466 municipalities), Southeast (90.2% of 1,668 municipalities) and South (96.6% of 1,191 municipalities). This difference was attributed to municipal infrastructure and the starting region of the survey, moving South to North.

**Fig. 1:**
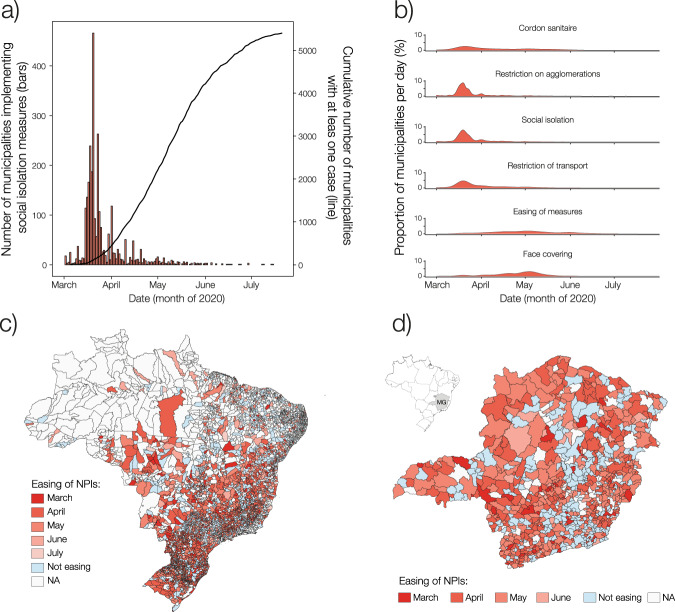
(**a**) Prohibition of non-essential services in the country (red bars) and the cumulative number of municipalities reporting at least one case (black line). (**b**) Density plots showing dates of adoption and easing of NPIs by municipalities in Brazil. (**c**) Starting month for easing of NPIs across municipalities in Brazil. (**d**) Starting month for easing of NPIs in the state of Minas Gerais (MG). NA - not applicable.

In March, a number of municipalities closed non-essential services (2,237), prohibited large gatherings (2,932), reduced public transportation (999), and implemented cordon sanitaires (930), with a rapid uptake by mid-March (Fig. 1a). At that time, COVID-19 cases were restricted to a few highly populated state capitals, with cases mostly associated with overseas travel^6^. Of the total of 3,958 mayors that responded to the question on implementing social isolation (closure of all non-essential services), 3,062 municipalities adopted the measure and among those, 2,738 (89.4%) implemented the measure before the first reported case in their municipality (Fig. 1b). Despite an early uptake and comprehensive NPI adoption, in only two months, SARS-CoV-2 spread from 296 municipalities (5.3%), on 31 March 2020, to 4,196 municipalities (75.3%), as of 31 May 2020 (Fig. 1a).

In other countries, implementations of NPI have been associated with fewer and delayed cases^7^; while a lack of coordination has been associated with disease spread and resurgence^8^. Although distancing measures were adopted across Brazil early in the pandemic, easing of these measures began as early as the end of March (Fig. 1c), often disregarding decisions by neighbouring municipalities, as illustrated in Fig. 1d for the state of Minas Gerais. We chose Minas Gerais to illustrate our dataset because of the high response rate to the survey. Of a total of 853 mayors in that state, only between 38–47 mayors failed to respond to specific NPI questions. We represented data absence with the colour white in Fig. 1d. We also chose to detail Minas Gerais because almost one-sixth of all Brazilian municipalities are located in that state. Municipal borders do not limit the flow for shopping trips or work commuting across towns^9,10^. Nevertheless, as Fig. 1c,d shows, decisions to ease NPIs were not coordinated between bordering cities.

## Contributions and Recommendations

When the Brazilian Supreme Court ruled on 15 April 2020 that mayors and governors were autonomous in their decisions related to the pandemic^11^, collecting local data on the management of the pandemic became urgent. With declining willingness of national and regional governments to impose national/regional lockdowns, the role of local measures to control the pandemic becomes increasingly important to understand transmission patterns at finer geographic scales. This high-resolution dataset on the NPIs in Brazil is an important contribution that also highlights challenges. Early and cohesive closure of non-essential activities was short-lived in Brazil, and municipalities are lifting distancing measures in an uncoordinated manner, starting as early as late March.

The easing of NPIs needs to be examined in relation to reductions in confirmed cases, hospital and testing capacities, mitigation policies such as compulsory use of face coverings, and the potential impact of local policies on neighbouring towns. City borders are porous and cities that have maintained strict social distancing policies may face a growing number of cases because of external decisions. Policy evaluation of Brazil’s management of the pandemic will need to account for the uneven duration of control measures through NPIs – which include personal (physical distancing, isolation quarantine, hand hygiene, and face covering), environmental (surface cleaning and ventilation) and social (travel restrictions, school and workplace closures, restriction on mass gatherings) – across the country^12^. This dataset allows for this assessment, aiding future research and policymaking.

## Methods

### Data collection

In order to collect these data, we started a specific collaboration with the Brazilian Confederation of Municipalities (CNM). The CNM is a non-partisan and non-profit organisation that works with mayors in Brazil, especially those that manage municipalities under 100,000 inhabitants, a focus that corresponds to 94% of Brazil’s municipalities. As the largest municipal association in Brazil, CNM possesses contact details of Brazilian elected mayors. The capillarity of that organisation makes it an ideal partner for such large-scale data collection. When the COVID-19 outbreak started and the CNM conducted the survey, we formed a partnership to analyse and deposit the dataset and thus expand access to these data. There were no shared financial responsibilities between researchers and the surveying institution.

The details of this cooperation were established through a meeting followed by a written agreement signed by the first and last authors of this paper with CNM on 9 April 2020. The partnership was established because of the need to understand the impact of decentralized measures in Brazil and what decentralisation causes to the spread of infectious diseases. Upon establishing this collaboration, CNM added further questions to the questionnaire for their monitoring of municipalities, such as budgetary information possibly affected by the pandemic. CNM had already designed and conducted a previous survey independently^13^, but upon our feedback, they added dates of implementation of NPIs to the survey questionnaire that we report on.

Mayors were contacted through a call centre. The CNM’s call centre is independently run; they contact mayors regularly on different policy themes. The phone-based survey collected information on local NPI policies related to COVID-19, Mayors had the option to receive a protected password to respond to the questionnaire online at a later time. When mayors were unable to respond to the survey questions, they suggested an alternative respondent, such as the municipal health secretary.

Mayors and representatives that responded to the survey had the option of updating previous answers: they were contacted by phone multiple times and they could use a protected password to update online. This methodology acknowledged cases when municipalities, in the course of data collection, could have relaxed NPIs to later re-establish social distancing. The questionnaire aimed at the first date of NPI implementation and the current state of easing NPIs. Short-lived decisions on NPIs during the course of the survey were not captured in the questionnaire. However, call centre operators wrote an observation on the limited cases when municipalities reported erratic NPIs lifting. A total of 144 municipalities (2.58% of the total) described having re-opened non-essential services due to local businesses’ and inhabitants’ pressure, and/or because of reduced number of confirmed cases, and/or they followed the state governor. However, in those 144 cases, they soon decided to re-establish social distancing when the number of confirmed cases increased. We detailed such municipalities in Online-only Table 1.

In total, the questionnaire had 47 questions; our database has 5 columns related to the identification of the municipality and 13 of the 47 questions that were part of our collaboration to document NPI policy strategies: 6 thematic questions with respective 6 dates of implementation and 1 question pertaining to percentage.

### Data classification

In summary, our dataset includes: (1) adoption of cordon sanitaire, (2) prohibition of agglomeration, (3) closure of all but essential services, (4) compulsory use of face covering, (5) reduction in public transportation services and if so, the percentage of the reduction, and (6) whether easing of the above measures were applied.

We had uniform data entry for all survey data. The dataset has information for the majority but not all municipalities in Brazil (4,027 of 5,568 municipalities and the Federal District). Below we offer a breakdown of the number of answers for each question: (Q1) adoption of cordon sanitaire: 3,976, (Q2) prohibition of agglomeration: 3,965, (Q3) closure of all but essential services: 3,958, (Q4) compulsory use of face covering: 3,952 (Q5) reduction in public transportation offer: 3,908, and (Q6) if there was already any easing of the above distancing measures, 3,947. When the answer to the above questions was yes, the column related to date of implementation was also populated.

We collected information on policies adapting a classification system on the 20 most frequent categories of NPIs to control the spread of COVID-19^4^. Considering the list of frequently adopted NPIs, we focused on the ones that would have a direct impact on the spatial mobility of residents. These measures were associated with a specific date of implementation. Adapting an international NPI classification to Brazil’s municipal reality requires some explanation. We did not report on closure of education institutions because such measures were implemented at the state level^3^. Similarly, we did not report on airport restrictions because such policies are not a municipal duty; on the other hand, the implementation of cordon sanitaire was a local decision and we report on those.

We reported on mass gathering cancellations (such as discotheques and sport events) and small gatherings restrictions (the closure of all but essential services). Reduction in public transportation services was not among the 20 most frequent NPIs categories^4^. However, this measure was frequently implemented, and potentially causing unintended consequences. Reductions in public transport services combined with low levels of social isolation can result in overcrowding of transport stations and vehicles. We invite further examination of transportation reduction and mobility patterns in Brazilian municipalities. Finally, we included a question on the compulsory use of face covering, as the compulsory use of masks is usually related to the re-opening of non-essential services and decrees were locally passed. For all NPIs, we also ask the date of implementation, and that field was populated in the format DD/MM/YYYY (Table 1). We also adapted the naming of NPIs to the Brazilian context, with mass gathering cancellation being named as “prohibition of agglomeration”, and small gathering cancellation as “closure of non-essential services”.

**Table 1:** Column names and data summary.

Column names (in Portuguese)	Column names (in English)	Number of records
IBGE	Unique Id	5569
Município	Municipality	5569
UF	State Acronym	27
Capitais	Capitals	Sim (27) / Não (5542)
Região	Region	5
Q1. Barreiras sanitárias (posto de monitoramento de entrada e saída de pessoas no Município)	Q1. Cordon Sanitaire (monitoring of entrance and exit of people in the municipality)	3976
Q1. Data Início (se sim)	Q1. Start date (if yes)	2021
Q2. Medidas restritivas para diminuição da circulação/aglomeração de pessoas.	Q2. Restrictions to avoid circulation/ agglomeration of people	3965
Q2. Data Início (se sim)	Q2. Start date (if yes)	3707
Q3. Medidas de isolamento social, permitindo APENAS serviços essenciais.	Q3. Measures of social isolation, allowing ONLY essential services	3958
Q3. Data Início (se sim)	Q3. Start date (if yes)	2901
Q4. Uso obrigatório de máscaras faciais.	Q4. Compulsory use of face covering	3952
Q4. Data Início (se sim)	Q4. Start date (if yes)	3588
Q5. Foram adotadas medidas de redução na oferta de transporte público?	Q5. Were any measures implemented to reduce the offer of public transportation?	3908
Q5. Qual foi a porcentagem de redução	Q5. What was the percentage of reduction?	1647
Q5. Data Início (se sim)	Q5. Start date (if yes)	1590
Q6. Houve flexibilização das medidas restritivas e de isolamento social.	Q6. Were measures of restriction and social isolation eased?	3947
Q6. Data Início (se sim)	Q6. Start date (if yes)	2319

During the 45 days of data collection (13 May to 31 July 2020), the CNM staff and the authors had access to a summary containing the total number of responses, which were classified as (1) complete, 3,174 interviews; (2) ongoing, 853 interviews; (3) pending, 1,536 interviews; or (4) without response, 6 interviews. We also received a partial dataset on the 13 of July.

Finally, for mapping NPIs (Fig. 1), we used the official spatial datasets with the administrative boundaries of states and municipalities organized by the Brazilian Institute of Geography and Statistics (IBGE).

## Data Records

The latest version of the data was updated on the 31 July 2020. We explain the time lapse between finishing the survey and making it available online below, which included budgeting time for validation. The dataset is available online (10.5061/dryad.vdncjsxs2)^14^. An additional data report and a description of the project (containing all 47 survey questions) are available online^15^. We describe the dataset fields that pertain to our cooperation in detail below. We have kept the dataset in its original language, Portuguese, to increase the usage by health professionals and scholars in Brazil. We offer a translation to English to make these data of international use:

### Main dataset

IBGE (unique_id): unique id for each municipality defined according to IBGE (Brazilian Institute for Geography and Statistics).

Município (Name of the municipality)

UF (State Acronym), defined as follows:

Acre – AC; Alagoas – AL; Amapá – AP; Amazonas – AM; Bahia – BA; Ceará – CE; Goiás – GO; Espírito Santo – ES; Maranhão – MA; Mato Grosso – MT; Mato Grosso do Sul – MS; Minas Gerais – MG; Pará – PA; Paraíba – PB; Paraná – PR; Pernambuco – PE; Piauí – PI; Rio de Janeiro – RJ; Rio Grande do Norte – RN; Rio Grande do Sul – RS; Rondônia – RO; Roraima – RR; São Paulo – SP; Santa Catarina – SC; Sergipe – SE; Tocantins – TO; Distrito Federal – DF;

Capital, dropdown option: sim (yes); não (no);

Região (Region), dropdown options as follows: Centro-Oeste (Centre-West); Norte (North), Sul (South), Nordeste (Northeast), Sudeste (Southeast);

Q1. Barreiras sanitárias - posto de monitoramento de entrada e saída de pessoas no Município - (Cordon Sanitaire - monitoring of entrance and exit of people in the municipality);

Q1. Data Início (Q1. Start date)

Q2. Medidas restritivas para diminuição da circulação/aglomeração de pessoas (Restrictions to avoid circulation/ agglomeration of people);

Q2. Data Início (Q2. Start date);

Q3. Medidas de isolamento social, permitindo APENAS serviços essenciais (Measures of social isolation, allowing ONLY essential services);

Q3. Data Início (Q3. Start date);

Q4. Uso obrigatório de máscaras faciais (Compulsory use of face covers);

Q4. Data Início (Q4. Start date);

Q5. Foram adotadas medidas de redução na oferta de transporte público? (Were any measures implemented to reduce the offer of public transportation?);

Q5. Qual foi a porcentagem de redução? (What was the percentage of reduction?);

Q5. Data Início (Q5. Start date)

Q6. Houve flexibilização das medidas restritivas e de isolamento social? (Were measures of restriction and social isolation eased?);

Q6. Data Início (Q6. Start date)

## Technical Validation

Interviewing mayors and health secretaries offers an important aspect of interpretation of laws and validation of dates related to non-pharmaceutical interventions. Even though decrees restricting physical contact or relaxing social distancing measures are available online, there were multiple laws on similar issues (e.g., a decree closing non-essential services followed by another one defining non-essential services, with a third one deciding on the duration of such activity). For that reason, consolidated information coming from those at the policy-making side increases precision.

To verify the correctness of received answers, we compared our dataset with existing information on adoption of NPIs on a city-level, which is mostly available for state-capitals. The eight capital cities for which we had data (Curitiba, Florianopólis, Fortaleza, Goiânia, João Pessoa, Manaus, Teresina, and Vitória), home to approximately 5% of the Brazilian population and distributed across all five Brazilian regions, offered answers and dates that are compatible with those collected by independent scholars looking at decrees^5^.

In addition to comparison with other datasets, we checked our data for possible erroneous entries, such as municipalities that eased NPIs before having implemented them. Five municipalities (Ajuricaba/RS; Estiva Gerbi/SP; Paiçandu/PR; São Lourenço/MG; Senador Cortes/MG) added 01/03/2020 as dates when they relaxed NPIs, which is equal or earlier than the date they implemented social distancing measures. These dates of NPI relaxation are likely to be erroneous.

During data collection, call centre operators detailed a limited number of municipalities that adopted NPIs, eased those, and later re-implemented distancing measures (as detailed in Online-only Table 1). The limited number of municipalities describing such behaviour corresponds with findings from other independently collected data on NPIs^5^. Researchers looking at capitals and second large cities found out that between NPIs implementation and easing, variations usually refer to capacity of specific services (e.g., restaurants or public transport) with cases of re-opening followed by a new set of distancing policies being still relatively rare. The reasons for such stable behaviour can vary and require further examination. This may be connected with the political costs of changing policies frequently, especially in a year of municipal elections. In addition to that, Brazil only significantly reduced its infection rate in October and at the time of writing, the country goes through an increase in number of infections. A new round of NPIs could potentially happen in the near future.

Finally, our multi-faceted data validation process also included broadly reporting on the data. On 9 September 2020, the CNM made public its summary report^15^ and we waited for two-weeks after that launch before submitting a first version of this dataset description. In this time period, no mayor requested revision of the data entered. Despite a lack of contestations from mayors to date, and the low number of possible erroneous data, omissions exist, and we invite users to complement this dataset using published decrees and media sources. The possibility of new lockdowns potentially followed by a new easing of NPIs would require a future survey.

Below we include examples of how these data could be used to allow for the continuity of policy surveys and guarantee a high level of cooperation between mayors and research units.

## Usage Notes

This database offers an opportunity for researchers and policymakers to examine the potential impacts of NPIs on COVID-19 transmission and control in Brazil. This is a unique dataset as it collates responses for the majority of all Brazilian cities, while previous datasets have mainly focused on capital cities, states, or have looked at a national level^4,5^. Because these data were collected through a survey, answers given by municipal authorities may be inaccurate. Unfortunately, even the scrutiny of laws also allows for a level of inaccuracy in interpretation and discrepancies on the exact date of policy implementation may be disputed in some cases. We have mitigated this problem with early and well-broadcasted release of a report on this dataset and technical validation included comparing our dataset with existing others on capital cities, we also looked for erroneous entries in our database. We invite researchers to use the dataset as it is. If significant inaccuracies are found, we will update our dataset and will describe such an update when it takes place.

This dataset represents a baseline for further research as it describes how COVID-19 response took place in a continental country such as Brazil. Because not all municipal authorities answered to all questions, particularly in the North region of Brazil, we suggest users to consider additional sources of information to document missing policy implementation, preferably using official sources such as local decrees. However, as decrees are not always available online, secondary sources such as media reports may need to be consulted.

A significant contribution of this dataset comes from the ‘release of NPIs’ column. At the time of writing, agglomerations (e.g., football stadiums) remain prohibited in most cities in Brazil. The easing of NPIs therefore mainly relates to the re-opening of non-essential services. Given that re-opening was asynchronous, researchers trying to retrieve exact dates on the easing of NPIs do not have a target period and searching for laws can be tiresome, especially when considering the total number of municipalities in Brazil. Finally, decrees that established the re-opening of shops and restaurants were often modified a few days later, such modifications especially related to capacity of small gatherings. The amendment of decrees makes NPI dates particularly susceptible to errors, potentially over or underestimating the date of easing of social distancing. A survey with mayors and health authorities is thus a fundamental tool as it allows us to listen to those on the frontline to describe when was the pivotal date for the reopening of services in town, information otherwise blurred in different decrees across time.

Scholars using our dataset could investigate whether easing of NPIs preceded increases in population mobility levels, or if adherence to NPIs was already low when NPIs were still in place. The connection between easing of NPIs and the compulsory use of face covering also invites further examination on the potential mitigation effects of masks. As the pandemic progresses and as Brazil is a highly affected country, we invite researchers to use the data to understand the pandemic and support health policymakers in their efforts.

## Online-only Table

**Online-only Table 1:** Municipalities reporting intermittent NPIs’ adoption during data collection, separated by region.

South	Southeast	Centre West	North	Northeast
Alfredo Wagner/SC	Alumínio/SP	Alto Horizonte/GO	Abaetetuba/PA	Acopiara/CE
Amaporã/PR	Américo de Campos/SP	Amaralina/GO	Altamira/PA	Alcântara/MA
Aurora/SC	Antônio Prado de Minas/MG	Barro Alto/GO	Alto Alegre dos Parecis/RO	Anajatuba/MA
Blumenau/SC	Arantina/MG	Carmo do Rio Verde/GO	Alto Paraíso/RO	Barreira/CE
Boa Vista da Aparecida/PR	Bandeira do Sul/MG	Colíder/MT	Alvorada/TO	Belmonte/BA
Canelinha/SC	Bilac/SP	Formoso/GO	Apuí/AM	Caridade do Piauí/PI
Cascavel/PR	Botelhos/MG	Glória de Dourados/MS	Bagre/PA	Cocal de Telha/PI
Chapada/RS	Botucatu/SP	Novo Horizonte do Sul/MS	Campos Lindos/TO	Fortim/CE
Hulha Negra/RS	Caputira/MG	Nova Veneza/GO	Dueré/TO	Gongogi/BA
Ibarama/RS	Cardoso/SP	Orizona/GO	Manoel Urbano/AC	Ipaumirim/CE
Jaboti/PR	Chiador/MG	Poconé/MT	Ponte Alta do Tocantins/TO	Maracás/BA
Jaguaruna/SC	Coronel Murta/MG	Ponta Porã/MS	Rio Crespo/RO	Monte das Gameleiras/RN
Lavras do Sul/RS	Córrego Danta/MG	Rio Verde de Mato Grosso/MS	Rio dos Bois/TO	Morpará/BA
Luiziana/PR	Cunha/SP	Santa Rosa de Goiás/GO	Urupá/RO	Muritiba/BA
Mandaguaçu/PR	Delfinópolis/MG			Palmeiras/BA
Minas do Leão/RS	Divisópolis/MG			Paulista/PB
Não-Me-Toque/RS	Dolcinópolis/SP			Ribeirão do Largo/BA
Passos Maia/SC	Dom Cavati/MG			Salinas da Margarida/BA
Pitanga/PR	Dona Euzébia/MG			Santa Rita de Cássia/BA
Porto Amazonas/PR	Espírito Santo do Pinhal/SP			Santana do Acaraú/CE
Rancho Alegre d’Oeste/PR	Felício dos Santos/MG			São José de Ribamar/MA
Rio dos Índios/RS	Fernando Prestes/SP			São Paulo do Potengi/RN
Salvador das Missões/RS	Frei Inocêncio/MG			Terra Nova/BA
Salvador do Sul/RS	Guarani/MG			Urandi/BA
Santa Clara do Sul/RS	Ibiraçu/ES			
São Miguel das Missões/RS	Igaraçu do Tietê/SP			
São Miguel do Iguaçu/PR	Indaiatuba/SP			
Tamboara/PR	Itapecerica/MG			
Toledo/PR	Itaúna/MG			
Tuneiras do Oeste/PR	Jaborandi/SP			
Ventania/PR	Jaguaré/ES			
Vila Nova do Sul/RS	Juiz de Fora/MG			
	Laranjal Paulista/SP			
	Lindóia/SP			
	Magda/SP			
	Marapoama/SP			
	Marechal Floriano/ES			
	Mococa/SP			
	Muzambinho/MG			
	Nacip Raydan/MG			
	Nova Resende/MG			
	Nova Venécia/ES			
	Patos de Minas/MG			
	Piquerobi/SP			
	Pitangui/MG			
	Praia Grande/SP			
	Presidente Juscelino/MG			
	Rio Espera/MG			
	Salmourão/SP			
	São Geraldo/MG			
	São João do Paraíso/MG			
	São João do Pau d’Alho/SP			
	São José dos Campos/SP			
	São Thomé das Letras/MG			
	Sardoá/MG			
	Seritinga/MG			
	Sumaré/SP			
	Taubaté/SP			
	Tupã/SP			
	Veredinha/MG			

The survey questionnaire focussed on first date of NPI implementation and latest state of NPIs’ easing. Details on irregular adoption of NPIs in the above municipalities can be found through local decrees at https://leismunicipais.com.br.
